# Laboratory quality management system fundamentals

**DOI:** 10.3389/fbioe.2025.1578654

**Published:** 2025-05-21

**Authors:** Segaran P. Pillai, Elizabeth Fox

**Affiliations:** Office of Occupational Safety and Health, Office of the Chief Scientist, Office of the Commissioner, United States Food and Drug Administration, Silver Spring, MD, United States

**Keywords:** quality assurance, quality management, LQMS, laboratory, implementation, total quality, continual improvement, workflow

## Abstract

A laboratory quality management system (LQMS) enables the effective operation of laboratories of all types and sizes. With rapid advances in technology (e.g., artificial intelligence and machine learning, advanced manufacturing) comes the need for laboratories worldwide to conduct proper change management and process improvement to meet the continued demand amidst major changes. In order to do so while ensuring that results and data are accurate, timely, and reproducible, it is crucial for laboratories to sustain a foundational LQMS that accommodates laboratory processes, document and records management, and a path for continual improvement in the laboratory itself and within its contextual organization. A foundational LQMS provides a framework to address gaps in process or product performance and risks present throughout the laboratory’s workflow, any of which could lead to a critical error that compromises the organization’s credibility. There are many LQMS frameworks–benchmarks such as consensus standards or regulations (e.g., Good Laboratory Practices for Nonclinical Laboratory Studies) – that the laboratory can select from to govern its LQMS. While these frameworks vary in applicability, there are several common elements across these frameworks that can serve as the basic components of any LQMS. The aim of this study is to review and assess 12 widely-recognized, fundamental aspects of an LQMS to identify actionable examples and templates that can enable effective implementation of a robust LQMS. A robust LQMS is one that fosters long term success of the laboratory, and which ultimately ensures reliable results, efficient operations, and the protection of public health.

## 1 Introduction: what is a laboratory quality management system?

**FIGURE 1:**
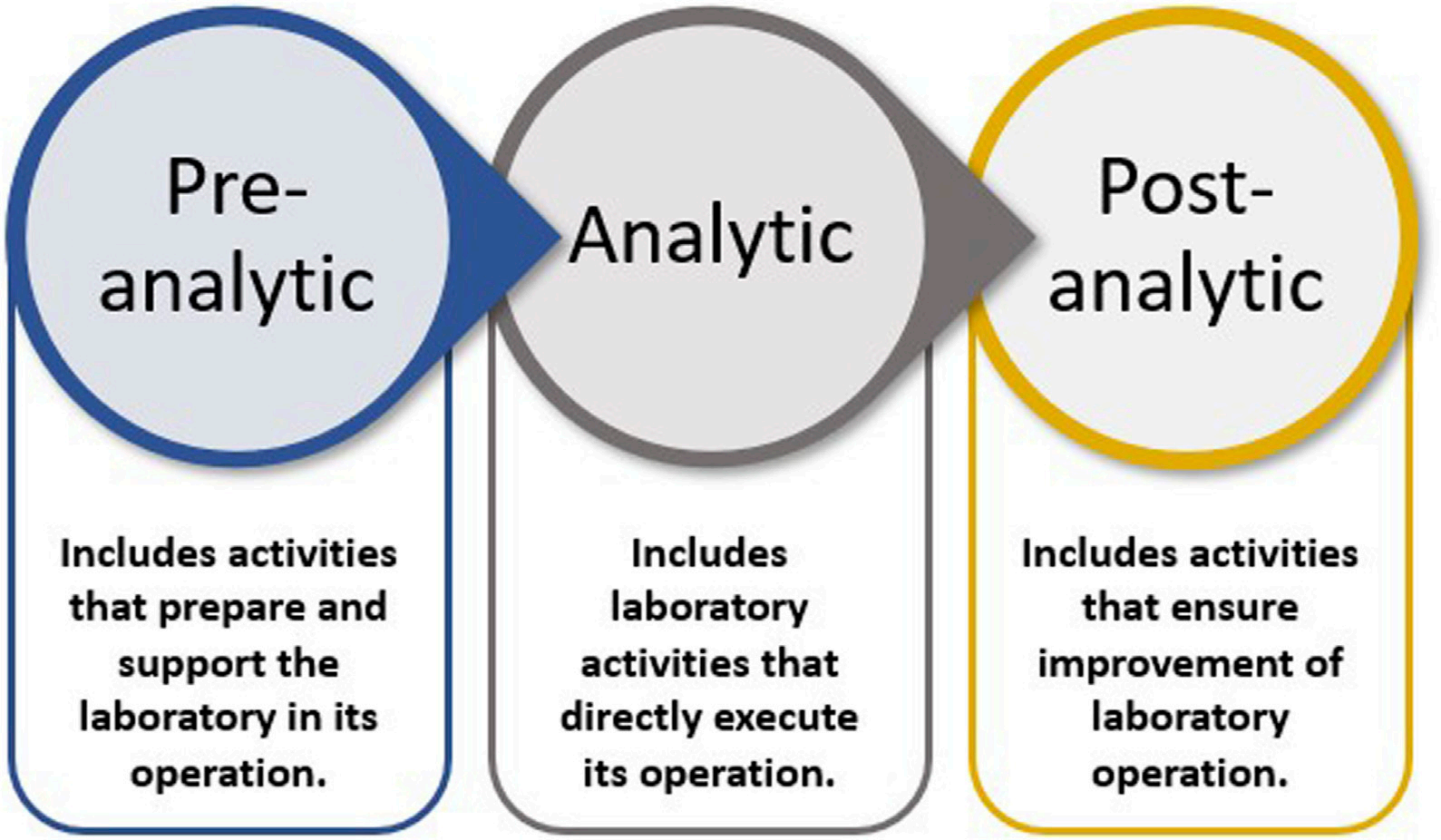
Phases of the laboratory path of workflow.

An LQMS is a formal system that documents the personnel, processes, and procedures by which laboratory management ensures the consistent quality of the processes conducted, outputs generated, and results reported. The entire set of operations that impacts a laboratory is considered the path of workflow. Workflow starts with the customer, ends with reporting, and results in interpretation, leading to appropriate actions and decisions. The path of workflow includes all steps before and after testing that affect laboratory outputs. There are three phases of the laboratory path of workflow: pre-analytic, analytic, and post-analytic (WHO, 2011), which Figure 1 graphically presents.

A *Nature* survey of 1,576 researchers found that 52% of responders agreed that there is a crisis of reproducibility; and over 70% have failed to reproduce another scientist’s data (Baker, 2016). To mitigate this crisis and protect public health through the generation of reliable and reproducible data, all aspects of laboratory operations, including organizational structure, processes, and procedures, must be addressed; an optimal solution for such is the implementation of a robust and efficient LQMS. To do so, consider establishing a quality policy and quality objectives; establishing processes, procedures, and systems to ensure that objectives and requirements are consistently fulfilled; and monitoring LQMS performance for continual improvement. To structure and guide effective LQMS implementation, activities can be grouped into components. Herein we describe one such framework, overviewed in the next section.

## 2 Overview of the 12 quality system essentials

**FIGURE 2:**
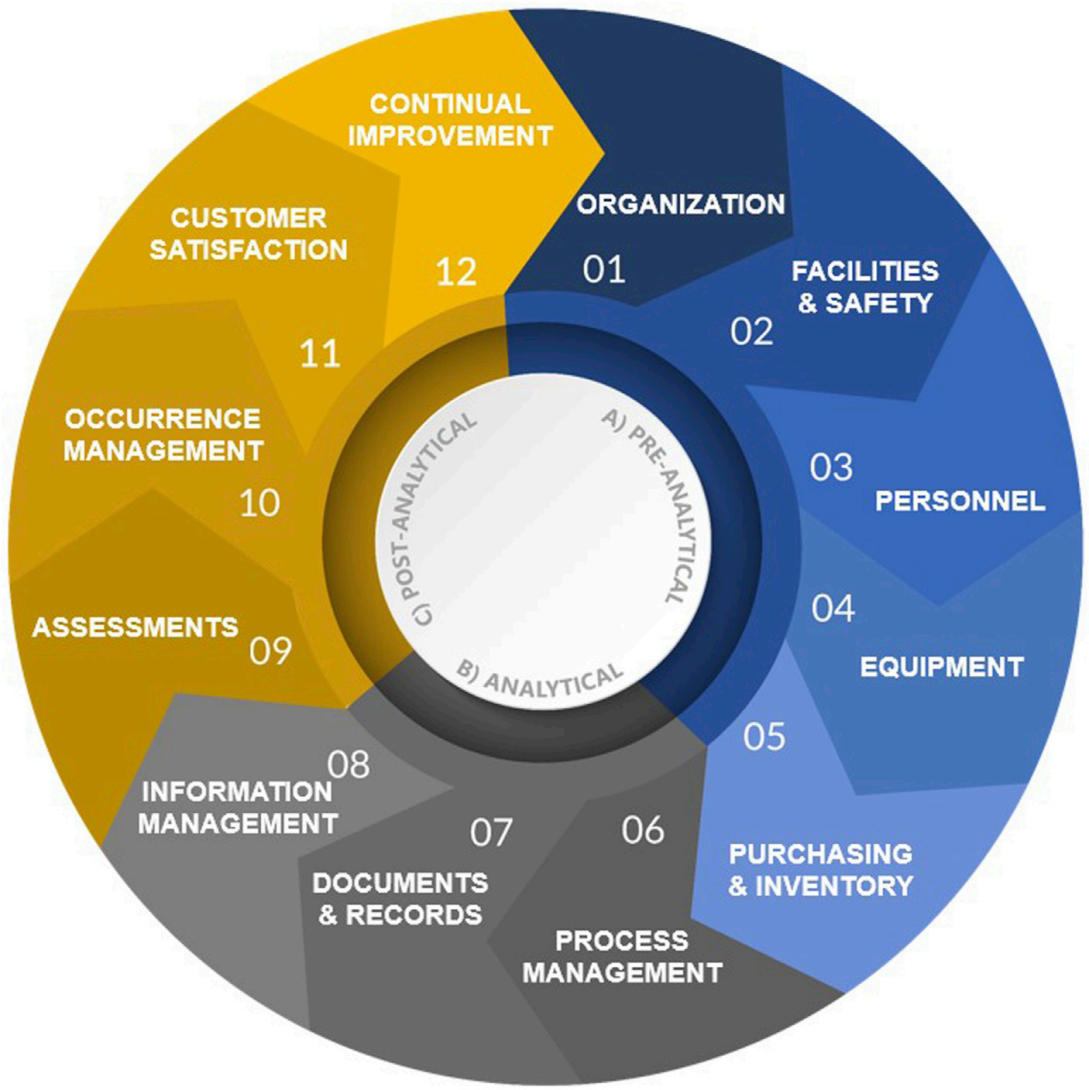
The 12 QSEs and their associated workflow phase.

The purpose of this article is to detail the fundamental elements of an LQMS and provide examples in support of implementation and improvement of organization-specific LQMS processes, procedures, and manuals that meet a laboratory’s unique needs. Fundamentally, an LQMS can be framed around 12 quality system essentials (QSEs), which are graphically represented in Figure 2 and aligned with the appropriate laboratory path of workflow. The 12 QSEs, modified from the World Health Organization’s (WHO’s) *Laboratory Quality Management System Handbook* (WHO, 2011), include Organization, Facilities and Safety, Personnel, Equipment, Purchasing and Inventory, Process Management, Documents and Records, Information Management, Assessments, Occurrence Management, Customer Satisfaction, and Continual Improvement. While very beneficial for understanding LQMS implementation, one limitation of the WHO Handbook is that it does not include templates for on-the-job use in the laboratory. The templates and examples provided herein (please see Table 1 and Supplementary Material) provide added benefit to laboratory personnel at all levels by enabling consistent documentation and a starting point for effective LQMS implementation.

**TABLE 1:** Examples of how to implement the QSEs.

QSE name	Implementation examples
1. Organization	• Develop and maintain an organizational chart(s) that include the laboratory staff; demonstrate how and where the laboratory sits within the overarching organization; and clearly convey the roles, responsibilities, purpose, and the laboratory’s reporting structure. See Supplemental Data S1 EXAMPLE_Laboratory Organizational Chart. • Establish detailed position descriptions for all laboratory roles, with clearly defined responsibilities and selection qualifications. See Supplementary Data S1: EXAMPLE_Laboratory Position Description. • Ensure that adequate resources are allocated for the laboratory to execute, monitor, and control the processes in the scope of the LQMS. • Establish clear communication channels and lines of authority within the LQMS to ensure effective messaging for awareness of quality activities and objectives, as well as empowerment of staff to provide suggestions for changes, process improvements, and other feedback (ideally carried out by leadership). • Develop and establish a quality manual - a quality manual can serve as a helpful tool to organize documented contextual elements of the LQMS (e.g., scope statement, quality policy and objectives), to provide comprehensive quality guidance for new laboratorians, and to aid assessors/auditors. • A responsibility assignment matrix known as a “responsible, accountable, supporting, consulted, and informed” (RASCI) matrix can be a helpful tool for outlining all the responsibilities associated with individuals’ roles involved for a given process. • Conduct management review of the LQMS at established intervals (minimally annual review is recommended). • Foster a culture of quality and responsible conduct of science that enables and motivates all laboratory staff to be proactive in meeting and exceeding quality expectations. Demonstrating a quality culture can be quite challenging for any organization; to provide some examples, this can include: o Designation of quality coordinators or liaisons to serve as “champions” of quality initiatives internal and external to the laboratory to increase awareness, ensure interdepartmental engagement, and promote collaboration. o Provision of informational “brown bag” or “lunch and learn” sessions for laboratory staff interested in learning more about laboratory quality management (QM) topics. o Establishment of dashboards visible to all employees that convey LQMS implementation progress and quality objective metrics. o Celebration of the laboratory’s successes and accomplishments of target goals and quality objectives. o Promotion of growth and development and continuing education opportunities for laboratory staff (see Section 5, Personnel). o Demonstration of how staff’s suggestions and opinions are valued and being addressed. o Provision of wellness programs for staff and promotion of a healthy work-life balance.
2. Facilities & safety	• Ensure that lines of communication related to safety and facilities are established (e.g., with a facilities manager and safety officer). • When designing laboratory workspace, ensure that workflow is optimized (coordinate with facilities staff, as appropriate). • Coordinate with facilities staff to ensure that laboratory move and renovation guides, and occupant emergency plans, are established. • Coordinate with safety staff to ensure that job hazard analysis is conducted for laboratory staff. o Ensure that laboratory staff are trained on hazards they handle on the job, as applicable (see Section 5, Personnel). • Coordinate with leadership, facilities, and safety staff, as appropriate, to ensure that the laboratory is secure and designed to enable safe and efficient operations (e.g., to prevent possible cross contamination in workflow areas). o Implement control of unauthorized access to the laboratories for sample security. o Ensure that the facility employs proper storage and environmental conditions for equipment, samples, testing areas, etc. as to not invalidate test results. o Ensure that environmental monitoring and evaluation procedures are established with the applicable equipment in place if conditions could impact the validity of laboratory results. See Supplementary Data S1: TEMPLATE_Environmental Sampling Plan. o Implement pest control in appropriate work areas. • Track all select agents and toxins used in the laboratory, as applicable, including the laboratorians permitted to handle them. • Make safety data sheets readily available (or coordinate with safety staff to do so, as appropriate) to all staff handling materials such as biological agents, toxins, chemicals, and reagents. • Establish procedures for laboratory housekeeping. • Coordinate with safety staff to ensure that the organization is fully compliant with all the rules and regulations pertaining to facility safety and health, through the development of safety aids such as fire plans, safety kits, spill kits, evacuation training, fire training, fire extinguishers, and related safety training. • Coordinate with safety staff to establish laboratory safety manuals, plans, guides, and templates, as appropriate. o Ensure that periodic safety inspections are conducted as per Occupational Safety and Health Administration 29 CFR 1960.25(c) (29 CFR 1960.25). (Occupational Safety and Health Administration, 1970)
3. Personnel	• Ensure that laboratory employees receive employee onboarding/orientation training that includes LQMS training (e.g., a basic orientation to principles of the quality system and how they as employees are responsible for quality in the organization). o Incorporate a safety orientation as part of the laboratory employee onboarding/orientation process as a helpful and engaging way to raise awareness of risks in the laboratory. • Provide on-the-job training for laboratory staff. • Retain records of staff competence (e.g., resumes, diplomas/transcripts, proficiency testing results) and update them periodically, as needed. • Evaluate actions taken to acquire additional/necessary competence; for example, mentor observation of job performance (if the mentor observes that the trainee can do the job effectively and safely, then the trainee can be considered competent) and annual performance reviews. • Ensure that all laboratory staff complete all required training in a timely manner. • Ensure that personnel with access to personally identifiable information (PII) data receive relevant training regarding data security and integrity, with records retained. • Establish training requirements for each role in the laboratory. o Ensure that staff receive and complete training requirements, including on approved procedures and laboratory processes per job descriptions (see Section 3, Organization); and retain and review/update training records. See Supplemental Data S1: TEMPLATE_On the Job Training Log. • Conduct competency assessments and/or ensure laboratorians participate in proficiency testing programs. • Provide continuing education and professional development opportunities to all laboratory personnel.
4. Equipment	• Develop/update a preventive maintenance procedure for laboratory equipment and keep documented maintenance logs (at established intervals) to provide evidence of fitness for purpose. This can be included within a broader equipment management procedure (see bullet below). • Develop/update an equipment management procedure to include: o Proper equipment identification, inventory, operation, status, and disposal/decommissioning. o Equipment labeling requirements (e.g., equipment identification number/serial number, location, last calibration date, next calibration due date). o Equipment qualification/validation (e.g., Installation Qualification, Operational Qualification, and Performance Qualification). o Equipment maintenance, including calibration service as applicable. • Create and implement a temperature log for every applicable piece of equipment in the laboratory that requires temperature to be within a specified range (e.g., incubators, refrigerators, water baths). • Develop/update a preventive maintenance log to keep track of completed and necessary routine equipment maintenance as part of the laboratory’s controlled document system. See Supplementary Data S1: TEMPLATE_Performance Indicator Development Form. o Include key details such as equipment identification details, a description of the maintenance conducted, the date conducted, and the subsequent due date for the maintenance item. o A monthly preventive maintenance checklist may be helpful for keeping track of items that can be prioritized based on risk level. • Calibrate, maintain, and certify all engineering controls to ensure the safety and health of the laboratorians.
5. Purchasing & inventory	• Conduct high-level resource budgeting/planning sessions at established intervals (typically carried out by leadership and finance personnel). • Coordinate with purchasing staff to establish requirements and procedures for ordering critical laboratory materials (e.g., reagents, microorganisms, *ex vivo* samples). • Identify and qualify suppliers for key laboratory materials to ensure suppliers are capable of meeting laboratory needs and requirements. o First, establish requirements for suppliers (e.g., proof of certification or accreditation to a specific standard that is important to the success of the laboratory). o Periodically re-evaluate these suppliers to ensure they continually meet requirements (see Section 11, Assessments). • Develop an inventory procedure for laboratory materials used for analysis such as chemicals, reagents, and other perishable items. It is recommended that the procedure include: o Requirement(s) for receipt and labeling of the material (including date received and expiration date). ⁃ A material receipt checklist can be a helpful aid to be used on the floor (in conjunction with the laboratory material receipt procedure). See Supplemental Data S1: EXAMPLE_Laboratory Material Receipt Checklist. o Requirement(s) for material inspection, including acceptance criteria. o Requirement(s) for safe and proper storage and handling (e.g., “first in, first out”), taking chemical compatibility into consideration for storage. o Requirement(s) for traceability to the purchase order and/or packing slip. o Requirement for signature from the custodial owner (as appropriate and applicable). o Requirement(s) for establishing intervals for conducting inventory checks in the laboratory. o Requirement(s) for proper disposal in accordance with environmental regulation. • Implement, maintain, and manage a robust laboratory inventory management system to enable staff to track all items in the laboratory and reduce the likelihood of ordering excessive supplies or having to trace an unmarked item (alternatively, develop and implement a laboratory material receipt form).
6. Process management	• Plan and document all laboratory activities, including laboratory management processes, laboratory support processes, and laboratory operational processes. o In initial stages of laboratory QM planning, it is helpful to create a turtle diagram that maps out a process to capture inputs, outputs, responsible parties, performance indicators, risks, etc. that are necessary for defining a laboratory protocol or procedure. Process maps/flow diagrams are also very useful tools that help to visually demonstrate a process. o Identify process owners when planning processes/services. • Define good documentation practices for the laboratory (see Section 9, Documents and Records). • Ensure that all laboratorians understand the laboratory path of workflow. • Establish/update quality indicators (also known as key performance indicators or quality objectives) for the laboratory. See Supplemental Data S1: TEMPLATE_Performance Indicator Development Form. • Establish a lot/batch numbering system for inputs (e.g., food samples for analysis) and outputs (e.g., certificates of analysis, reports), as applicable. • Develop/update procedures for protecting the integrity of regulatory samples for analysis (e.g., if cold storage is required for samples, build that into the procedure with the acceptable temperature range, along with a requirement for removal of all cardboard boxes to prevent mold growth). • Establish written specifications for laboratory outputs and outline them clearly (preferably built into the forms, protocols, etc. that laboratorians use to record and/or report data so that in-specification and OOS results can be easily identified or differentiated). • Apply QC materials to laboratory analyses for each batch of samples tested to ensure test accuracy and identify contamination; these materials may include Certified Reference Materials, Reference Materials, replicate analysis, positive/negative control samples, laboratory control samples, blanks, and matrix spikes. Ensure the QC material is suitable for the test; for example, for enumeration assays, use a quantified control material. • Leverage visual workplace aids (e.g., posters, system alerts) to remind laboratorians to apply required engineering and administrative controls within the applicable work area.
7. Documents & records	• Develop written procedures for all laboratory processes. Critical processes should be identified and standardized to ensure laboratory quality and result integrity. See Supplemental Data S1: TEMPLATE_SOP. • Identify external documented information used within the scope of the LQMS (e.g., reagent certificates of analysis, equipment manuals). • Establish and follow a records management program, including retention periods and disposition. • Establish a document control procedure that includes instruction for identification, development, review, and approval; distribution; access, retrieval, and use; storage and preservation (including legibility); control of changes (e.g., version control); retention; and disposition for internal and external documented information in the scope of the LQMS. • Review documented information and ensure that controlled documents (e.g., laboratory procedures, templates, forms, reports, notebooks) have all adequate controls according to the procedure established. • Transition paper records to electronic system(s) that provide adequate access, security, and preservation, as applicable and as resources allow. • Implement and routinely update a master list of all forms used by the laboratory. • Maintain documents and records in a secure, access-controlled location. • Ensure that all instrumentation records, calibration records, maintenance records, certification records, training records, and proficiency testing records are maintained. • Ensure that updated documents, procedures, and policies are readily available to staff as either hard copies or electronic versions, with previous versions archived.
8. Information management	• Train all laboratory staff on the applicable IM systems used by the laboratory, including the importance of keeping laboratory data confidential as needed. • Ensure the correct level of access permissions are applied to all laboratory personnel. • When planning for a major shift to a new information or documents/records management system, it will be important to track file inventory to ensure that all items are properly migrated and able to be tracked and located. See Supplemental Data S1: TEMPLATE_Data Migration & Inventory Map. • Ensure that data is stored using servers that are well equipped to handle the organization’s needs, including the appropriate storage capacity, the availability of subject matter experts (e.g., information technology staff), security updates and patches, data backups, and recovery. o Establish a data management procedure that describes the above aspects of the information management process and the intervals at which updates and backups should occur in order to protect data security and integrity and prevent data loss. • Ensure that stored data, reports, and other laboratory information are traceable and readily retrievable. • Ensure that appropriate governance is applied to laboratory data that falls under intellectual property agreements as applicable (e.g., Material Transfer Agreements). • Maintain data integrity over its entire lifecycle.
9. Assessments	• Establish procedures for planning for, follow-up actions for, and appropriate employee conduct during, external assessments (e.g., proficiency testing, certification audits). • Establish procedures for conducting audits of suppliers. • Establish procedures for conducting internal LQMS audits (i.e., self-assessments) at intervals, including a requirement to develop audit reports to be shared with leadership. o Providing resources for QA staff to continue professional development, such as for the attainment of quality assurance or QM certifications, could better enable staff to conduct successful internal audits. o Ensure impartiality with auditing and assessments (it is recommended that auditors do not assess their own work areas). • Define quality indicators for monitoring/assessing LQMS activities (see Section 8, Process Management). • Implement an assessment findings tracker, which can assist with management of corrective actions (CAs) related to assessment findings. See Supplemental Data S1: TEMPLATE_Assessment Findings Tracker.
10. Occurrence management	• Establish a Risk Management procedure, to include: o How risks are to be identified, categorized (e.g., by impact and severity) and tracked with actions to address them. See Supplemental Data S1: EXAMPLE_Occurrence Management Workflow. o The interval at which risk assessments are to be conducted. o When and how to review actions to address risks; and the effectiveness of the actions (such as annual management review). o Preferred risk management strategy or specific organizational risk assessment model that is to be used for planning of actions to address the risks identified – the following treatment approach options are an example: ⁃ Avoid: Eliminate the threat by eliminating the cause. ⁃ Mitigate: Identify ways to reduce the probability or the impact of the risk (e.g., develop a contingency plan, implement a control point to monitor results). ⁃ Accept: This refers to the conscious decision to live with the consequences of the risk and the results of the potential loss. No action is planned. ⁃ Transfer: Assign another party as responsible for the risk (e.g., outsource external resources). o How risks specifically related to impartiality and confidentiality are to be treated, including any requirements to eliminate or minimize threats (risks) to impartiality (as applicable). • Establish a nonconformance (NCM) and CAs procedure that includes: o Definition of responsibilities involved with NCM management, including authorization of resumption of work, and who is responsible. o How NCM must be identified (e.g., labeled, flagged in a laboratory information management [IM] system), documented, and segregated from conforming outputs. o Categorization or classification of NCM according to relative severity, potential for adverse impacts, risks and/or other factors. o The requirement to conduct and document risk assessment and impact analysis for identified NCM, and the preferred methodology for conducting this evaluation. o Decision-making guidance on the need for action and timeframes. o The requirement to retain records of all NCM and associated actions; and how the records are to be kept. o Actions that may be taken when NCM is identified, including halting work, recall of proficiency testing items and/or reports, and conducting formal CA when recurrence is possible or there is doubt regarding compliance with procedure. Action should be aligned with the significance determined from the risk assessment and impact analysis. o How to conduct root cause analysis, when this should be done, and any necessary cross-functional collaboration needed. o Implementation of CAs. o How and when to review CA effectiveness, how it must be documented, and when a CA is permitted to close out (recommended to have ample time before closure to assess if there is true recurrence prevention). • Conduct root cause analysis (e.g., Failure Modes & Effects Analysis, Ishikawa diagram] to help identify negative outcomes of a process and possible weaknesses in existing controls. Root cause analysis tools can be used when assessing an existing process, redesigning a process, planning improvement goals, or analyzing failures of a process. • Develop a CA plan when an occurrence (e.g., NCM) is identified to document and manage the occurrence and its associated actions to address it.
11. Customer satisfaction	• Identify all internal and external customers (i.e., interested parties), and document their needs, expectations, and product/service requirements (including agreements). • Develop/update a customer feedback process; a written procedure is recommended, which could stand alone or be built into a broader customer service procedure (see appendix for an example customer feedback process workflow). See Supplemental Data S1: EXAMPLE_Customer Feedback Process Workflow. • Gather customer feedback at established intervals via feedback intake forms, customer portals, and/or surveys. o To distribute surveys, an online form system (e.g., Microsoft Form, Survey Monkey) could be used with links submitted via a client distribution list. o Once data is gathered, evaluate the degree of satisfaction (e.g., use built-in online form system data analytics to provide insights). • Track and manage all customer complaints and feedback, including any corrections and/or CAs taken (see Section 12, Occurrence Management). • Incorporate customer satisfaction as a performance indicator for the laboratory, and an item as part of the periodic review and evaluation of LQMS effectiveness (see management review in Section 3, Organization).
12. Continual improvement	• Establish quality indicators for overall LQMS effectiveness (see Section 8, Process Management). • Document, plan, and implement opportunities for improvement (OFIs). See Supplemental Data S1: TEMPLATE_Lessons Learned Log • Implement and routinely update a lessons learned log to track lessons learned through all phases of a project or initiative as OFIs. • If changes to the LQMS must be made, ensure they are conducted in a planned and formalized manner, which can include revising processes/procedures in the scope of the LQMS, revising training materials, adding items to a communication plan, establishing new monitoring/measurement, etc. • Establish continual improvement as an item to be included for review and evaluation during management review (see Section 3, Organization). • Increase staff participation through empowerment and through rewards and recognition. Rewards and recognition ideas include: o Implementing a “quality badge” that is awarded to any employee that identifies an NCM, OFI, or other problem that affects the quality of laboratory results and data or the LQMS.

Implementing the 12 QSEs is beneficial for many laboratory types with the goal of assuring the continued generation of accurate, reliable, reproducible, and timely data. Pillai et al. reviewed five widely recognized quality management (QM) regulations and standards commonly applied in laboratories and found that at least 10 out of 12 QSEs (83%) are addressed in all five frameworks (Pillai et al., 2022). This finding highlights the significance of QSEs in laboratory QM. The subsequent sections provide descriptions and recommendations pertaining to each QSE, beginning with QSE 1: Organization.

## 3 QSE 1: organization

The first QSE, Organization, focuses on the laboratory staffing structure and how it sits within its organization. Leadership’s commitment plays a critical role in the successful implementation of an LQMS. If leadership does not clearly demonstrate interest in adherence to the LQMS, it will likely become difficult to instill a strong culture of quality in the laboratory. Designing a laboratory’s organizational structure in a manner that ensures quality objectives are met and aligned with organizational strategy can serve to fulfill its mission. For example, the role of a “Quality Liaison” can be established to uphold a culture of quality and promote best practices and policies. Implementation of the Organization QSE would also ensure that roles and responsibilities for monitoring and assessment are established to mitigate risks. With the appropriate resources and leadership’s dedication to the success of an LQMS, a laboratory is further equipped to protect public health.

## 4 QSE 2: facilities and safety

Facilities and Safety involves the laboratory environment and the processes, procedures, and plans that support its safe and secure operation. With this QSE, laboratories follow approved facility procedures, instructions, rules, national and state regulations, guidelines, and standards. A suitable environment for effective and conformant laboratory operations should be provided and maintained, including social, psychological, and physical factors that ensure staff safety and health. Consider the purpose and type of laboratory work when determining what is needed; for example, if environmental monitoring is conducted, ensure that proper facility systems are in place (e.g., air filtration, humidity monitoring, alarms for out-of-range measurements). Some may wonder, what does safety have to do with quality? While quality and safety practices have slightly different goals, they are complementary. Quality is defined as the “degree to which a set of inherent characteristics of an object fulfills requirements” (ISO, 2015a), and safety is an important laboratory requirement. It is recommended that safety and quality staff have separate, clearly delineated responsibilities, but also collaborate to achieve objectives. Leadership is responsible for establishing policies and resources to ensure safety and health of employees, the organization’s most critical asset. It is everyone’s duty to embrace safety and responsible behavior in their work. Regardless of the type of laboratory, a higher safety risk must inform enhanced safety protocol. Implementing this QSE is a proactive measure to sustain safety and quality in the laboratory.

## 5 QSE 3: personnel

The Personnel QSE focuses on defining job descriptions, qualifications, and training requirements for laboratory staff. This begins before they are hired–establishing competence requirements and position descriptions ensures that qualified staff conduct laboratory operations–and continues with enabling staff to grow professionally. Laboratory staff should receive appropriate training upon hiring and throughout employment, including safety training aligned with applicable regulatory requirements; hands-on training on laboratory procedures and equipment; and quality training needed for LQMS activities. Personnel must be fully trained and deemed competent to execute their responsibilities safely and accurately prior to beginning work. According to a 2019 review, a survey of 2,400 academic researchers found that while 70% received safety training, only 26% were trained within 30 days of onboarding; among other harrowing statistics presented, one study found that 25% of researchers had not been trained in the specific hazard with which they worked (Ménard and Trant, 2020). Hazard identification and risk mitigation strategies are foundational components of LQMS. To ensure training effectiveness, laboratory management should conduct competency assessments and/or proficiency testing at established intervals. This may reveal the need for additional or specific training, and incorporating safety requirements into assessments will help ensure that nothing is missed when it comes to the laboratory’s most important resource: its people.

## 6 QSE 4: equipment

Well-maintained equipment is crucial to safe laboratory operation and the quality of data generated. The Equipment QSE focuses on ensuring that policies and procedures are in place and available to appropriately receive, identify (label), qualify, inventory, calibrate, and maintain critical equipment throughout its lifecycle, including a decommissioning/retirement policy. Critical equipment refers to laboratory equipment or instrumentation used for scientific operations (e.g., sample analysis, processing, handling, and/or manipulation and data collection). It is important for leadership to prioritize equipment management and allocate resources to ensure it is done effectively. Routine preventive maintenance and calibration of critical equipment will help to ensure the validity of results. A preventive approach will not only minimize downtime from malfunction, but it can also reduce the likelihood of variability in test results, which can occur over time as equipment gradually degrades. Testing positive, negative, and calibration controls on analytical equipment, where applicable, ensures proper function. For equipment used to store valuable materials and conduct analysis—the loss of which can be detrimental—it is essential to implement redundant systems to manage equipment failure risks. Equipment must also be fit for purpose; fitness for purpose can be determined in many ways (e.g., calibration), but evidence should be documented (e.g., calibration records, temperature records). When traceability is required or essential, it becomes critical to identify (e.g., label), calibrate using measurement standards (e.g., National Institute of Standards and Technology), and safeguard equipment from anything that would invalidate it for use. If measurement equipment is found to be unfit (e.g., control results out-of-specification), the laboratory should determine if previous results that the equipment generated are valid or invalid, and take appropriate action as necessary (e.g., repeating analysis using newly calibrated equipment, recalling data). The above activities should be documented as evidence that they were performed–for example, an equipment receipt form may be completed when new equipment arrives, and a preventive maintenance log could be updated when routine service is performed (with certificates and receipts of service saved).

## 7 QSE 5: purchasing and inventory

Maintaining inventory and monitoring the quality of suppliers’ products and services helps ensure consistent quality of results. This QSE focuses on processes and procedures to appropriately receive, identify, use, store, and discard materials. Routine inventory should be conducted to ensure that chemicals, reagents, and other materials are within expiry and properly stored. Inventory management can prevent occurrence of testing interruptions (see Section 12, Occurrence Management). It can also assist with measurement of stock levels over time to optimize stock quantities of fast- and slow-moving materials. Another key aspect of this QSE is ensuring purchased material fitness-for-use. Establishing procedures and criteria for supplier qualification and continued capability, and coordinating with purchasing staff to implement guardrails (e.g., approved vendor lists), ensure that the laboratory receives suitable materials that support the validity and reproducibility of results. Additionally, handling requirements should be incorporated into procedures, including signing for receipt, confirming if the material is hazardous, inspecting for damage, and storing properly. For testing and research materials—the integrity of which is critical to scientific conduct—it is essential to manage risks associated with inventory such as incorrect storage, misidentification, and retention past expiry.

## 8 QSE 6: process management

Process management involves organizing and controlling laboratory processes and procedures to ensure accurate and reliable testing, which ultimately ensures the quality of data produced and the accurate interpretation of results. This QSE describes the planning, managing, and documenting of connected processes. Well-planned and managed processes help a laboratory to be more effective and efficient. This QSE involves establishing quality control (QC) activities. The purpose of QC is to monitor processes related to the analytic phase of testing and to allow for detecting errors in testing so that corrective action can be taken before flawed results are released from the laboratory. Variability in manufacturing processes is the result of many disruptions that occur during their implementation and by their nature cannot be 100% eliminated (Misztal and Ratajszczak, 2025). This natural variability further increases the need for QC. Whether the laboratory science is quantitative or qualitative, automated or manual, employing adequate QC procedures ensures the reliability of results generated. For a laboratory associated with product or method development, it is advisable to incorporate appropriate verification and validation procedures when implementing a new product or method or a significant change to an existing product or method.

## 9 QSE 7: documents and records

The Documents & Records QSE focuses on the development, control, and maintenance of written laboratory policies, processes, procedures, protocols, work instructions, forms, and records associated with laboratory operations. Records are documented information that serves as evidence of laboratory activities (e.g., laboratory notebooks, reports). Key aspects of analysis or research such as instrument used, maintenance/calibration data, and reagents used (including manufacturer, date of receipt, date of expiration, lot number, etc.), should be documented to support the reproducibility of results. Employees should be able to find documents and records when needed and easily understand the information conveyed. Document control procedures should ensure that applicable documents are identifiable; access-controlled; reviewed and approved before distribution and at periodic intervals; archived or destroyed as applicable; and that audit trails are in place to ensure accountability for edits or corrections (i.e., capturing who, when, and why, without obscuring the original entry). Procedures should also address correct format and media; storage and preservation; change control; and retention and disposition. It is recommended to maintain records electronically to the greatest extent possible. Records of evidence of conformity (i.e., records that prove requirements have been met), as well as non-conformity, should be protected from unauthorized alterations.

## 10 QSE 8: information management

The Information Management (IM) QSE focuses on management of data and its flow through the laboratory, from incoming to outgoing. IM systems may be paper-based, electronic, or a combination of both; whatever technology is employed, the IM QSE is essential and is closely related to the Documents and Records QSE (WHO, 2011). The main difference is that the IM QSE encompasses a broader system for management of information (e.g., security, data governance, how information moves through the path of workflow), while the Documents and Records QSE focuses on controlling written information about policies, processes, and procedures, and the completed information that serves as records (e.g., a batch record, a form that was filled out). The IM QSE involves reviewing and meeting information requirements (e.g., intellectual property, material transfer agreements) and disseminating information (e.g., test results) in approved system(s) to end users in a secure, timely, and accurate manner. Properly addressing this QSE ensures that data is accurate and confidential, and accessible to authorized users. Laboratories should establish procedures to ensure that information received, generated, disseminated, and published is effectively governed to safeguard its quality, integrity, reproducibility, security, and confidentiality based on sensitivity and/or privacy. Leadership (or designee) should assign access levels for laboratory personnel per job descriptions and work requirements, and for external users. Stored data, reports, and other information should be traceable and readily retrievable.

## 11 QSE 9: assessments

The Assessments QSE is a way to analyze an LQMS’s efficacy through internal and external assessments (e.g., audits), as well as through performance evaluation in an external quality assessment (EQA) program (Dhara et al., 2024) to verify conformance to regulatory, accreditation/certification, and customer requirements. The QSE involves evaluating laboratory performance compared to a standard, benchmark, or performance of other laboratories, as appropriate. Assessments and audits may be internal (e.g., self-assessments conducted within the organization by personnel trained for assessment, belonging to a department separate from the one being assessed) or external (e.g., conducted by a third-party entity). An internal assessment, for example, could be a process audit conducting by QA staff for the manufacturing department; and an external assessment could be an ISO/IEC 17025:2017 accreditation audit (International Organization for Standardization, 2017) for a calibration laboratory conducted by an ISO registrar. Standardized assessments conducted at established intervals help to evaluate the effectiveness of the LQMS. Properly addressing this QSE ensures comprehensive implementation of the LQMS, supports identification of opportunities for improvement, and improves the ability to achieve quality objectives. To implement this QSE, laboratories should establish procedures for internal assessments. Quality assurance (QA) staff (or appropriate designee) should also develop and implement procedures for supporting external assessments. Further, QA staff/designee(s) should define quality indicators for LQMS assessment.

## 12 QSE 10: occurrence management

In the complex world of laboratories, occurrences can and will naturally occur in any phase of the path of workflow. An occurrence is defined as any event that has a negative impact on an organization, including its personnel, the product of the organization, equipment, or the environment in which it operates (WHO, 2011). Unintended errors and other events in the laboratory can have serious consequences that affect the quality of its results and could negatively impact public health or trust. But when occurrences are properly managed through identification, reporting using appropriate channels, and application of corrective actions (CAs), the output of occurrence management is a continually improving organization. This QSE involves establishing procedures to investigate unintended consequences (e.g., non-conformances) from laboratory activities, identify root causes, and implement CAs to eliminate recurrence. Laboratories cannot prevent every error or incident, and events should be tracked to identify process gaps. But properly addressing this QSE can reduce risk of future occurrences, personnel injury, facilities damage, equipment or material loss, and events adverse to organizational decisions. Laboratory management should establish procedures for: identifying, reporting, documenting, and investigating occurrences; conducting risk assessments associated with occurrences; developing and implementing risk mitigation strategies; and implementing CAs properly with effectiveness checks.

## 13 QSE 11: customer satisfaction

The Customer Satisfaction QSE emphasizes laboratory customers, their expectations, and the importance of designing process(es) to meet those expectations. To uphold a laboratory’s organizational and public health missions, it is essential to consider the needs and expectations of its customers (i.e., interested parties). Customers can be both internal (e.g., leadership, another department that the laboratory interacts with such as manufacturing) and external (e.g., clients, regulators, proficiency testing providers). Anyone impacted by laboratory outputs could be considered a customer. Ultimately, the laboratory generates a product—data and results—for its customers. If the customer is not well served, the laboratory is not achieving its primary function. This QSE involves procedures to monitor customer needs through feedback mechanisms (e.g., surveys, emails, online chat), quality indicators, and assessments (see Section 11, Assessments). Leadership is responsible for ensuring that customer expectations are understood and met. Properly implementing this QSE can reduce customer dissatisfaction, ensure that the “voice” of the customer is heard, and improve internal processes through addressing feedback; it also demonstrates commitment to the customer. To do so, laboratory management should establish procedures to identify internal and external customers; document their needs, expectations, and requirements; manage satisfaction through feedback review and analysis; and address complaints in a timely manner.

## 14 QSE 12: continual improvement

The Continual Improvement QSE focuses on increasing LQMS effectiveness and efficiency to provide added benefit to the organization and its customers. Improvement strategies such as the Plan-Do-Check-Act approach, defined below, could be used to continually evaluate and improve laboratory processes:

Plan: establish the objectives of the system and its processes, and the resources needed to deliver results in accordance with requirements; identify and address risks and opportunities;
Do: implement what was planned;
Check: monitor and measure processes (as applicable) and the resulting outputs against objectives, requirements, and planned activities, and report the results;
Act: take actions to improve performance, as necessary (ISO, 2015b).

In support of organizational missions and the protection of public health, it is essential to take steps to implement and improve upon the LQMS to facilitate increased accuracy, reliability, reproducibility, and timeliness of data. Ensuring the quality of laboratory outputs is the main objective of an LQMS and ultimately relies on continual improvement. This QSE involves procedures to monitor and evaluate the effectiveness of the LQMS. Review of internal assessment reports, occurrences, customer satisfaction surveys, and data trends, are all ways to continually improve the laboratory. Leadership, QA staff, and laboratory management are responsible for monitoring LQMS compliance and evaluating the system overall for effectiveness. Implementing this QSE can help to identify and reduce risks in the laboratory, as well as increase productivity by fixing process inefficiencies; additionally, it enhances commitment to a culture of quality and responsible conduct of science.

## 15 Discussion

The foundational information described in this article and implementation examples provided in Table 1 support effective laboratory QM related to the 12 QSEs in the framework outlined. It is important that organizational leadership, laboratory management, QA staff, and laboratory staff have a foundational understanding of the measures available to ensure quality of laboratory operations, including the LQMS QSEs. Application of the QSEs can not only organize and improve the LQMS implementation process, but it can also ensure that all aspects of an LQMS are addressed to mitigate risks, allow for structured assessments, and prepare for third-party certification or accreditation. This study, including provision of examples and templates pertaining to each key area described, demonstrates that implementing a well-structured, robust framework that the 12 QSEs offer can support the continued success of the laboratory using consistent, standardized documentation and a clear understanding of what implementation could entail–this is critical for planning the LQMS. Implementation using the supplementary templates provided can also contribute to time and cost savings.

As laboratories conduct different types of work and have distinct needs and goals, further examination of the QSEs beyond this article may be beneficial, including identifying how to modify and apply their respective concepts to reflect a laboratory’s chosen LQMS framework (e.g., Good Laboratory Practices for Nonclinical Studies [GLP]; ISO standards) in order to improve applicability.

Table 1 below provides illustrative examples of how staff responsible for oversight of the LQMS, and leadership where appropriate, could apply each QSE within the organization to demonstrate conformance, should the organization decide to conform to the 12 QSE framework.

## Abbreviations

CA, Corrective Action; CFR, Code of Federal Regulations; EQA, External Quality Assessment; GLP, Good Laboratory Practices; IEC, International Electrotechnical Commission; IM, Information Management; ISO, International Organization for Standardization; LQMS, Laboratory Quality Management System; NCM, Nonconformance; OFI, Opportunity for Improvement; PI, Principal Investigator; QA, Quality Assurance; QC, Quality Control; QM, Quality Management; QSE, Quality System Essential; WHO, World Health Organization.
