# Supplementary material for: Laboratory quality management system fundamentals
Source: Front Bioeng Biotechnol. 2025 May 21;13:1578654. doi: 10.3389/fbioe.2025.1578654 (PMC12133829; doi:10.3389/fbioe.2025.1578654)
Supplement: Supplementary file 1 [file DataSheet1.zip › Supplementary Materials/EXAMPLE_Customer Feedback Process Workflow.pptx]

## Slide 1
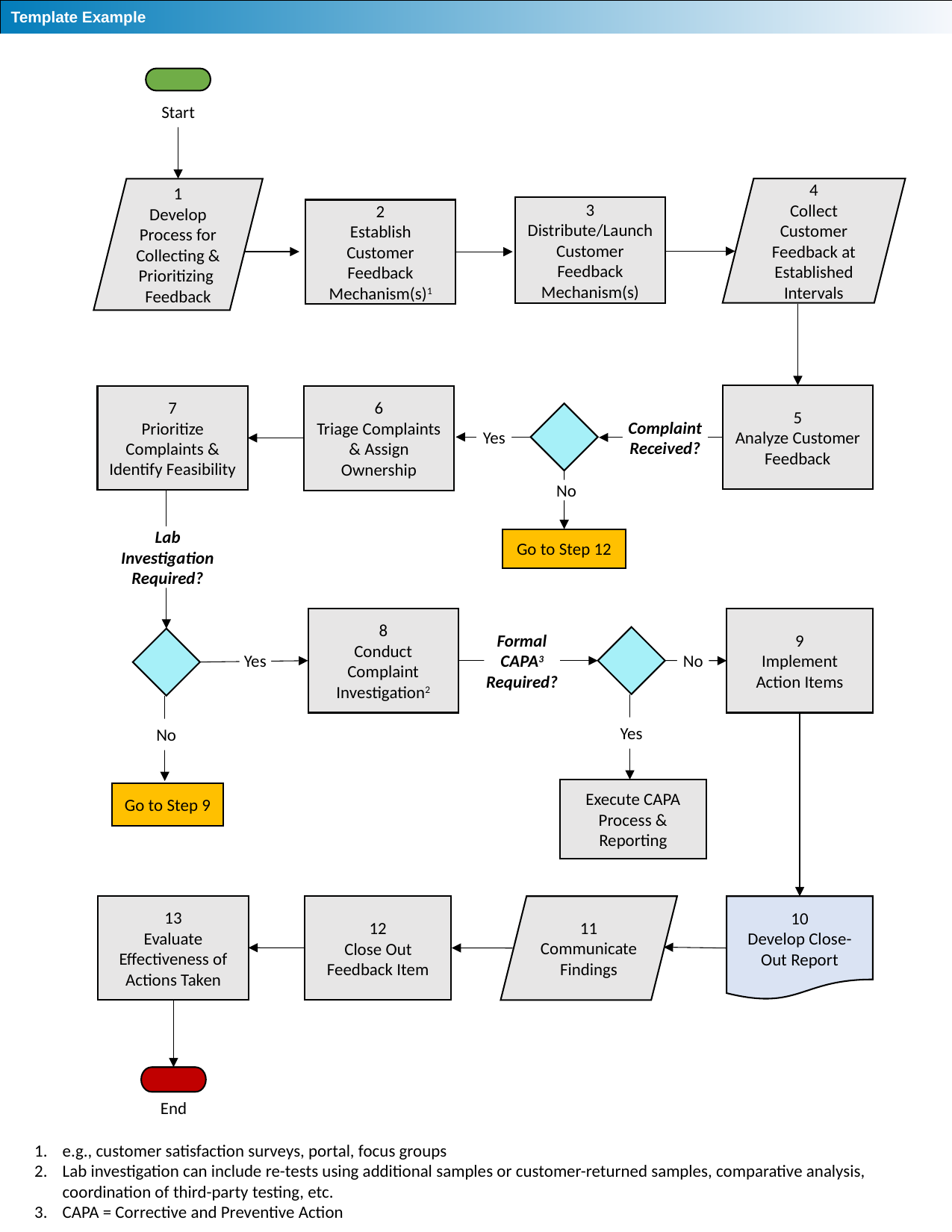

Template Example
Start
4
Collect Customer Feedback at Established Intervals
1
Develop Process for Collecting & Prioritizing Feedback
3
Distribute/Launch Customer Feedback Mechanism(s)
2
Establish Customer Feedback Mechanism(s)1
5
Analyze Customer Feedback
7
Prioritize Complaints & Identify Feasibility
6
Triage Complaints & Assign Ownership
Complaint Received?
Yes
No
Lab Investigation Required?
Go to Step 12
8
Conduct Complaint Investigation2
9
Implement Action Items
Formal CAPA3 Required?
No
Yes
Yes
No
Execute CAPA Process & Reporting
Go to Step 9
11
Communicate Findings
10
Develop Close-Out Report
13
Evaluate Effectiveness of Actions Taken
12
Close Out Feedback Item
End
e.g., customer satisfaction surveys, portal, focus groups
Lab investigation can include re-tests using additional samples or customer-returned samples, comparative analysis, coordination of third-party testing, etc.
CAPA = Corrective and Preventive Action

## Slide 2
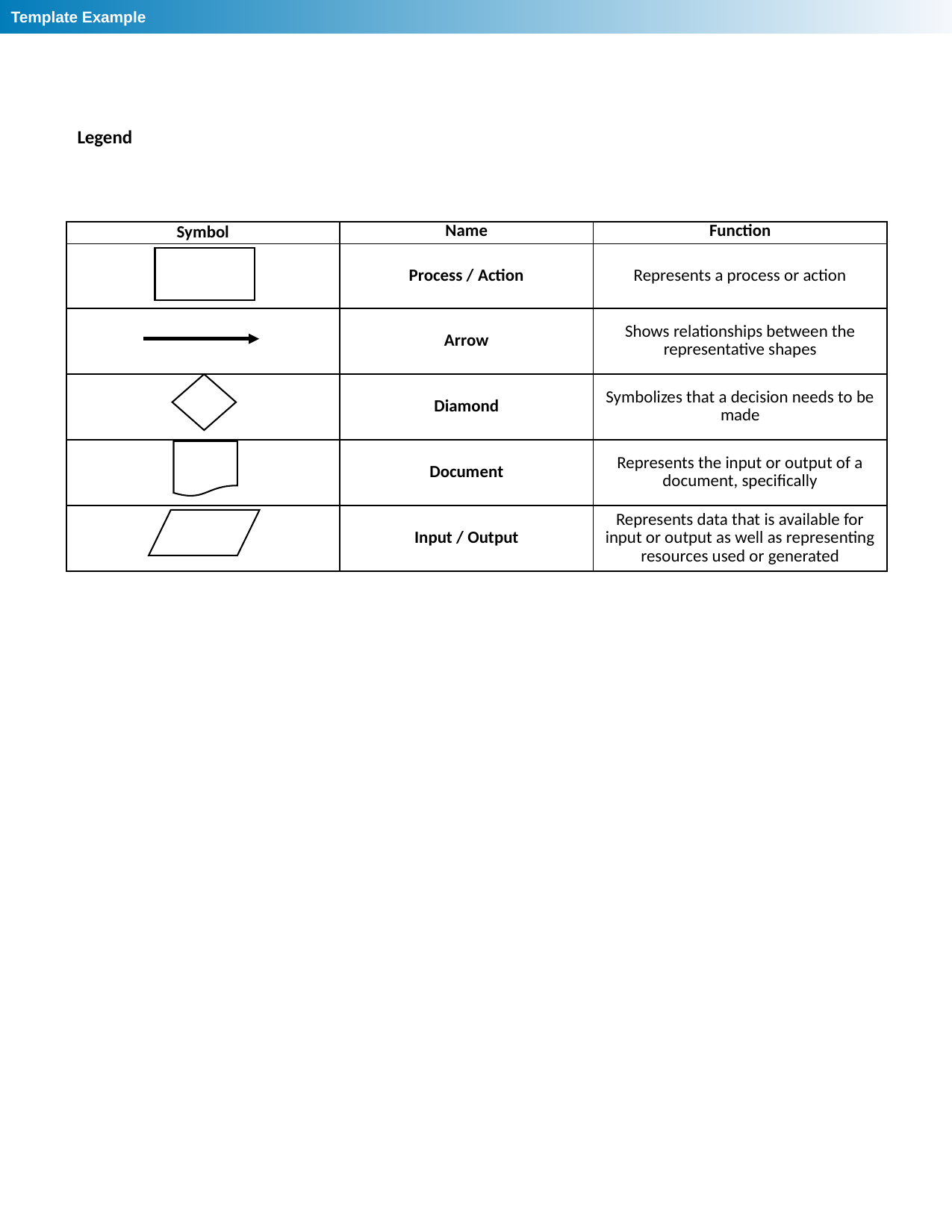

Template Example
Legend
| Symbol | Name | Function |
| --- | --- | --- |
| | Process / Action | Represents a process or action |
| | Arrow | Shows relationships between the representative shapes |
| | Diamond | Symbolizes that a decision needs to be made |
| | Document | Represents the input or output of a document, specifically |
| | Input / Output | Represents data that is available for input or output as well as representing resources used or generated |
