# Supplementary material for: Laboratory quality management system fundamentals
Source: Front Bioeng Biotechnol. 2025 May 21;13:1578654. doi: 10.3389/fbioe.2025.1578654 (PMC12133829; doi:10.3389/fbioe.2025.1578654)
Supplement: Supplementary file 1 [file DataSheet1.zip › Supplementary Materials/EXAMPLE_Laboratory Material Receipt Checklist.docx]

**Laboratory Material Receipt Checklist**

**Introduction**: [Insert text informing the user of the purpose and scope of the checklist (for example, for critical reagents or biological materials for laboratory analysis, or customer samples received for testing) and/or links to the applicable standard operating procedure (SOP) or work instruction (WI) that specifies use of the checklist].

| Receiver Name (First, Last): |  | Package Receipt Date: | Click to enter a date. |
| --- | --- | --- | --- |
| Vendor: |  | Item Category: | [insert drop-down list of applicable types of items] |
| Order #: |  | Item Lot #: |  |
| Product Name: |  | Quantity: |  |
| Item Catalog #: |  | Item Storage Location: |  |

**PLEASE USE THE “COMMENTS” BOXES TO PROVIDE ADDITIONAL INFORMATION, IF NEEDED**

**A. Shipment and Handling**

| # | Question* | Yes | No | N/A |
| --- | --- | --- | --- | --- |
| 1 | Example question: Did the package arrive in good condition and undamaged? |  |  |  |
| 2 | Example question: Was the correct item shipped per the purchase order? |  |  |  |
| 3 | Example question: Are the packing slip and item in agreement? |  |  |  |
| 4 | Example question: Were shipping requirements met by the carrier (e.g., correct temperature, without packing peanuts) |  |  |  |
| 5 | Example question: Were all applicable safety and hazard labels observed and understood by the receiver? |  |  |  |
| 6 | Example question: Is the item intact (e.g., no visible evidence of leakage, cracks, tears, broken components)?  ***Note****: if “no”, and the material is hazardous, seek supervisory guidance on the appropriate spill clean-up and disposal protocol.* |  |  |  |

| *If “No” was selected for any of the questions in Section A, please elaborate on the issue below: |
| --- |
|  |

| Comments |  |
| --- | --- |

**B. Material Acceptance Criteria**

| # | Question | Y | N | N/A |
| --- | --- | --- | --- | --- |
|  | *Section Applicable?* |  |  |  |
| 1 | Example question: If a Certificate of Analysis (COA) is required for the item, was it provided by the vendor as either an electronic or physical copy? |  |  |  |
| 2 | Example question: If a COA is required for the item, does it pass all required specifications? |  |  |  |
| 3 | Example question: Does the item pass visual inspection per [insert supporting procedure, work instruction, product specification sheet, etc.]? |  |  |  |
| 4 | Example question: If in-house acceptance testing is required for the item, does it pass all required specifications per [insert supporting procedure, work instruction, product specification sheet, etc.]? |  |  |  |
| 5 | Example question: If the item is a chemical, reagent, or hazardous material, is a material safety data sheet provided by the vendor as either an electronic or physical copy? |  |  |  |

| Comments |  |
| --- | --- |

**C. Inventory & Storage**

| # | Question | Y | N | N/A |
| --- | --- | --- | --- | --- |
| 1 | Example question: If the item is a chemical, reagent, test sample, biological, or hazardous material, did the receiver properly label it per [insert supporting procedure, work instruction]? |  |  |  |
| 2 | Example question: Did the receiver store the item in its proper location and according to storage requirements? |  |  |  |

| Comments |  |
| --- | --- |

**D. Attachments**

| # | Question | Y | N | N/A |
| --- | --- | --- | --- | --- |
|  | Example question: Are copies of the following documents attached to this checklist: |  |  |  |
| 1 | Packing slip? |  |  |  |
| 2 | Purchase order? |  |  |  |
| 3 | COA from vendor? |  |  |  |
| 4 | In-house acceptance test results? |  |  |  |
| Comments |  | | | |
