# Supplementary material for: Laboratory quality management system fundamentals
Source: Front Bioeng Biotechnol. 2025 May 21;13:1578654. doi: 10.3389/fbioe.2025.1578654 (PMC12133829; doi:10.3389/fbioe.2025.1578654)
Supplement: Supplementary file 1 [file DataSheet1.zip › Supplementary Materials/EXAMPLE_Laboratory Organizational Chart.pptx]

## Slide 1
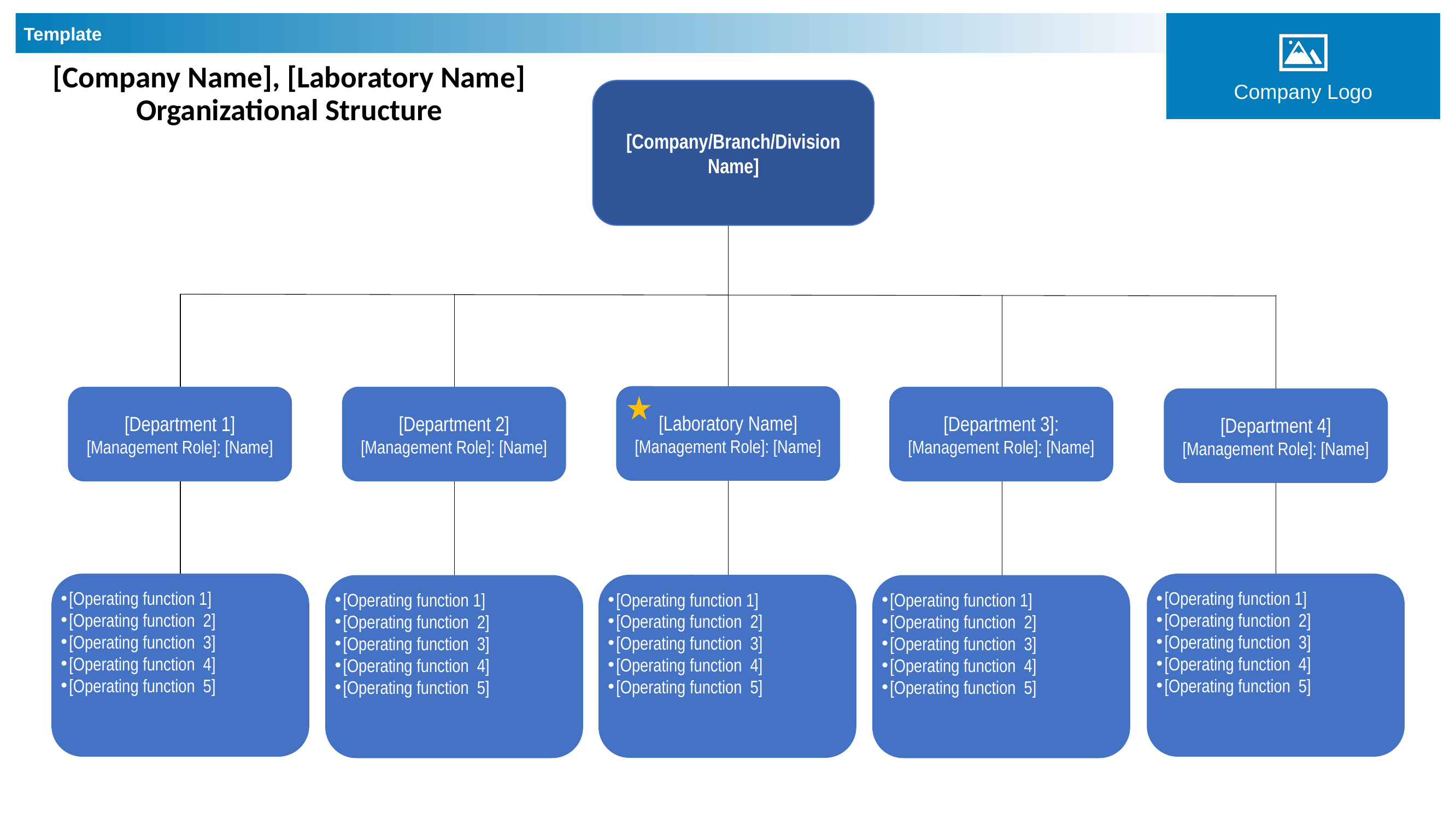

Company Logo
Template
# [Company Name], [Laboratory Name] Organizational Structure
[Company/Branch/Division Name]
[Laboratory Name]
[Management Role]: [Name]
[Department 3]:
[Management Role]: [Name]
[Department 2]
[Management Role]: [Name]
[Department 1]
[Management Role]: [Name]
[Department 4]
[Management Role]: [Name]
[Operating function 1]
[Operating function 2]
[Operating function 3]
[Operating function 4]
[Operating function 5]
[Operating function 1]
[Operating function 2]
[Operating function 3]
[Operating function 4]
[Operating function 5]
[Operating function 1]
[Operating function 2]
[Operating function 3]
[Operating function 4]
[Operating function 5]
[Operating function 1]
[Operating function 2]
[Operating function 3]
[Operating function 4]
[Operating function 5]
[Operating function 1]
[Operating function 2]
[Operating function 3]
[Operating function 4]
[Operating function 5]

## Slide 2
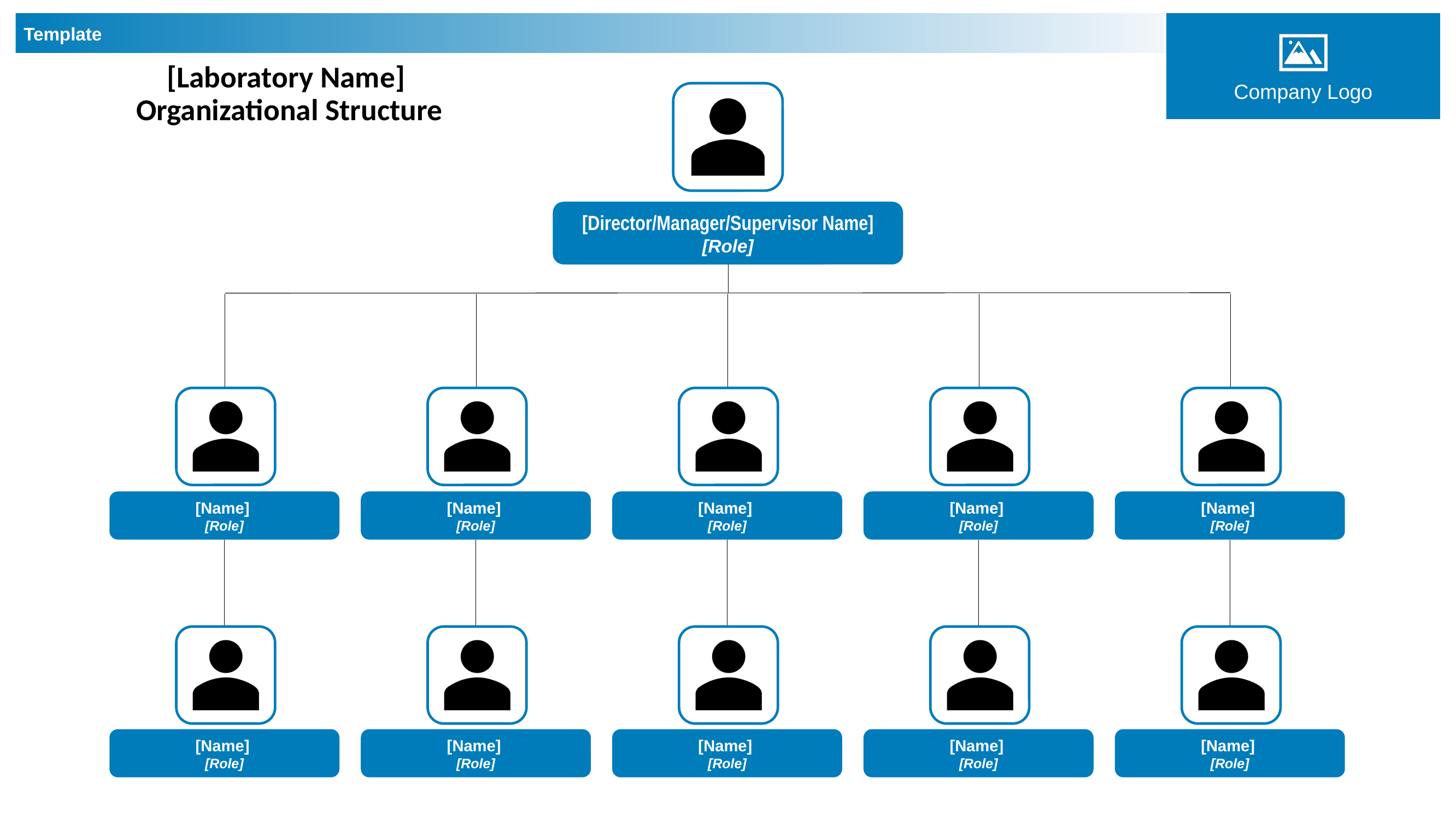

Company Logo
Template
# [Laboratory Name] Organizational Structure
[Director/Manager/Supervisor Name]
[Role]
[Name]
[Role]
[Name]
[Role]
[Name]
[Role]
[Name]
[Role]
[Name]
[Role]
[Name]
[Role]
[Name]
[Role]
[Name]
[Role]
[Name]
[Role]
[Name]
[Role]

## Slide 3
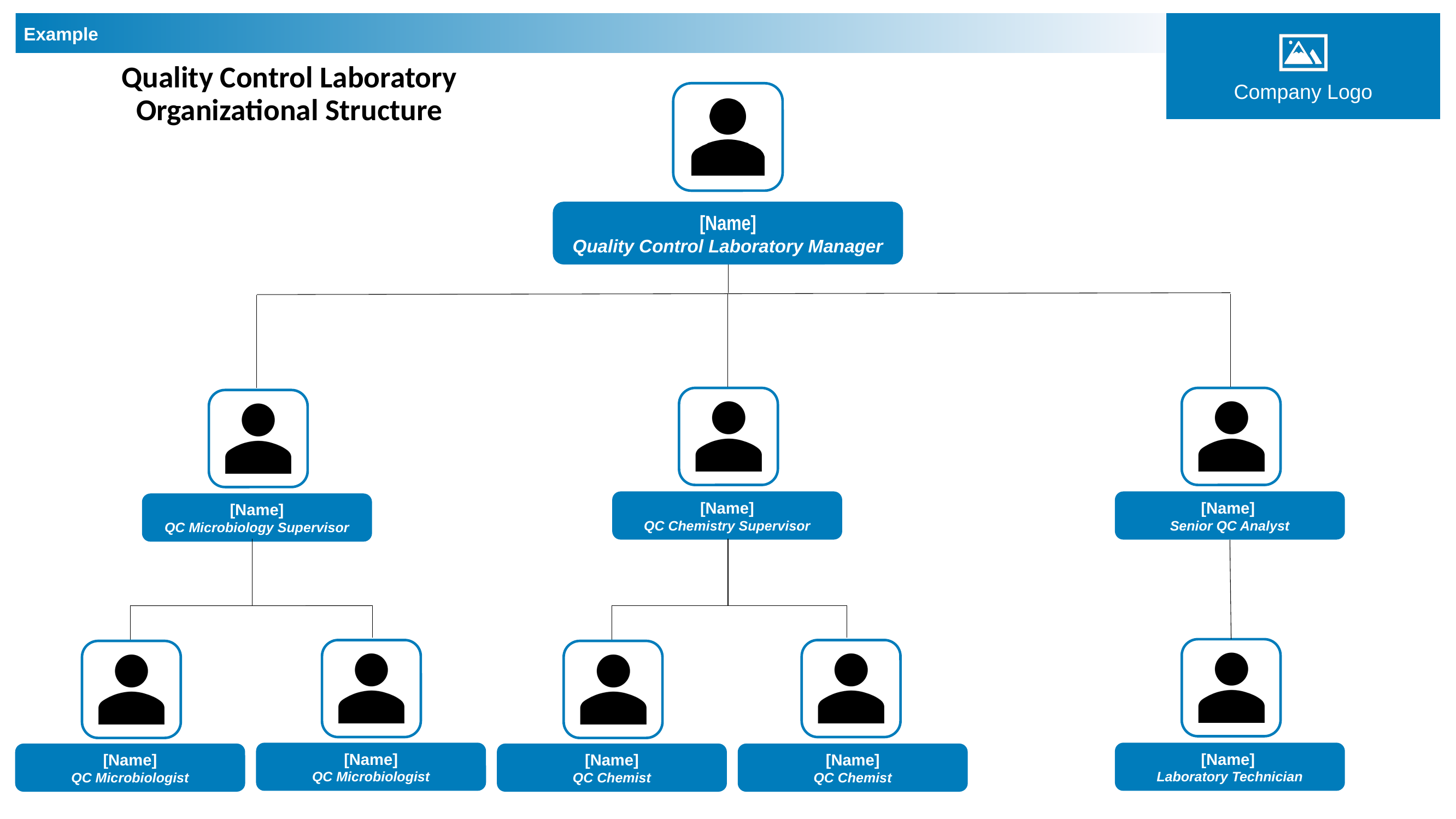

Company Logo
Example
# Quality Control Laboratory Organizational Structure
[Name]
Quality Control Laboratory Manager
[Name]
QC Chemistry Supervisor
[Name]
Senior QC Analyst
[Name]
QC Microbiology Supervisor
[Name]
QC Microbiologist
[Name]
Laboratory Technician
[Name]
QC Microbiologist
[Name]
QC Chemist
[Name]
QC Chemist
