# Supplementary material for: Laboratory quality management system fundamentals
Source: Front Bioeng Biotechnol. 2025 May 21;13:1578654. doi: 10.3389/fbioe.2025.1578654 (PMC12133829; doi:10.3389/fbioe.2025.1578654)
Supplement: Supplementary file 1 [file DataSheet1.zip › Supplementary Materials/EXAMPLE_Laboratory Position Description.docx]

**Laboratory Quality Control Analyst Position Description**

| **Introductory Statement:** | This position is located in the Quality Control Laboratory of Organization X in Akron, Ohio. The incumbent is primarily responsible for the environmental monitoring of state county water samples in accordance with the Ohio Environmental Protection Agency (EPA) regulations, and Standard Methods for the Examination of Water and Wastewater. The Quality Control (QC) Analyst conducts microbial and chemical analysis and additional duties to support the QC program and reports results, while adhering to Good Laboratory Practices (GLP). The incumbent also conducts activities in support of the goals, objectives, and priorities of the laboratory quality management system (LQMS) program at Organization X, to include continual improvment of the quality of the LQMS program. The incumbent upholds a culture of total quality and exemplifies excellent customer service throughout Organization X. |
| --- | --- |
| **Position Type** | Full-time (40 hours per week), Monday – Friday; First Shift |
| **Major Duties:** | - Responsible for executing microbiological standard methods procedures using aseptic technique, including Fecal Coliform, *E. coli*, *Enterococci*, Total Coliform, and Heterotrophic Plate Count (HPC). - Responsible for executing standard methods in wet chemistry including ion chromatography, Biochemical Oxygen Demand (BOD)/Carbonaceous BOD (CBOD), Dissolved Oxygen, Total Phosphorus, Orthophosphorus, Turbidity, Total Residual Chlorine, pH, Conductivity, Total Hardness, and Alkalinity. - Responsible for good documentation practices. - Responsible for reporting results on the plant waste water to the Operations Director. - Responsible for reporting county water sample results to Supervisor, including identified deviations and nonconformances. - Responsible for aseptic potable and waste water sampling. - Responsible for daily maintenance of the laboratory’s housekeeping including cleanliness, routine equipment preventive maintenance, maintaining organization, and non-routine equipment troubleshooting. - Responsible for carrying out assigned duties in a manner consistent with the safety policies defined by Organization X as well as state and local environmental regulations. Reports safety hazards, potentially hazardous situations, incidents, and close calls to the supervisor. Assists the supervisor to identify and eliminate unsafe conditions. - Performs other duties as assigned. |
| **Physical Demands:** | Walking; standing; sitting; bending; lifting items up to 20 lbs. |
| **Work Environment:** | The work involves moderate risks in conducting laboratory operations and requires special safety precautions, e.g., working around moving parts, carts, or machines; and exposure to microbiological organisms or irritant chemicals. Employees will be required to use personal protective equipment including lab coats, steel-toed boots, goggles, and gloves. |
