# Supplementary material for: Laboratory quality management system fundamentals
Source: Front Bioeng Biotechnol. 2025 May 21;13:1578654. doi: 10.3389/fbioe.2025.1578654 (PMC12133829; doi:10.3389/fbioe.2025.1578654)
Supplement: Supplementary file 1 [file DataSheet1.zip › Supplementary Materials/EXAMPLE_Occurrence Management Workflow.pptx]

## Slide 1
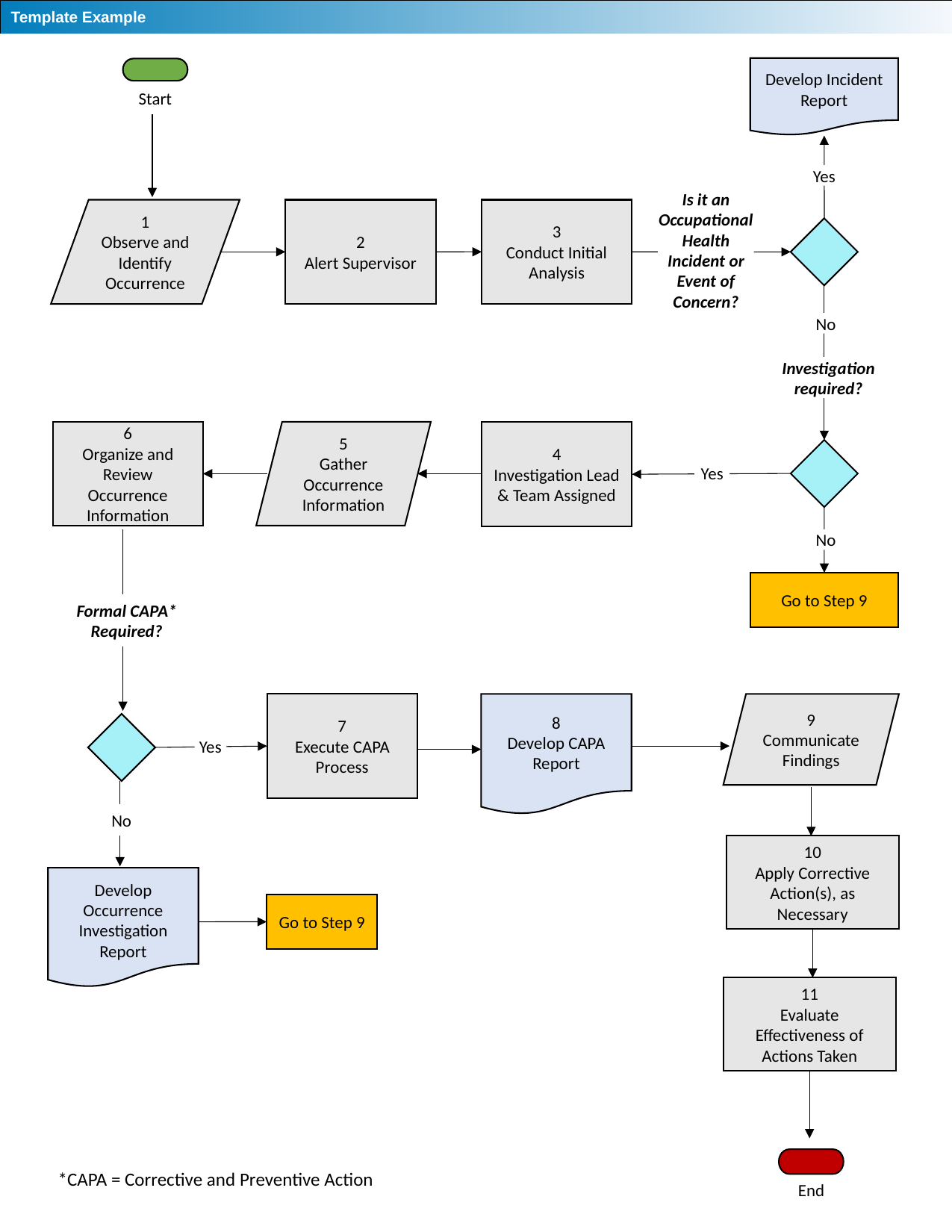

Template Example
Develop Incident Report
Start
Yes
Is it an Occupational Health Incident or Event of Concern?
1
Observe and Identify Occurrence
2
Alert Supervisor
3
Conduct Initial Analysis
No
Investigation required?
5
Gather Occurrence Information
6
Organize and Review Occurrence Information
4
Investigation Lead & Team Assigned
Yes
No
Go to Step 9
Formal CAPA* Required?
7
Execute CAPA Process
8
Develop CAPA Report
9
Communicate Findings
Yes
No
10
Apply Corrective Action(s), as Necessary
Develop Occurrence Investigation Report
Go to Step 9
11
Evaluate Effectiveness of Actions Taken
*CAPA = Corrective and Preventive Action
End

## Slide 2
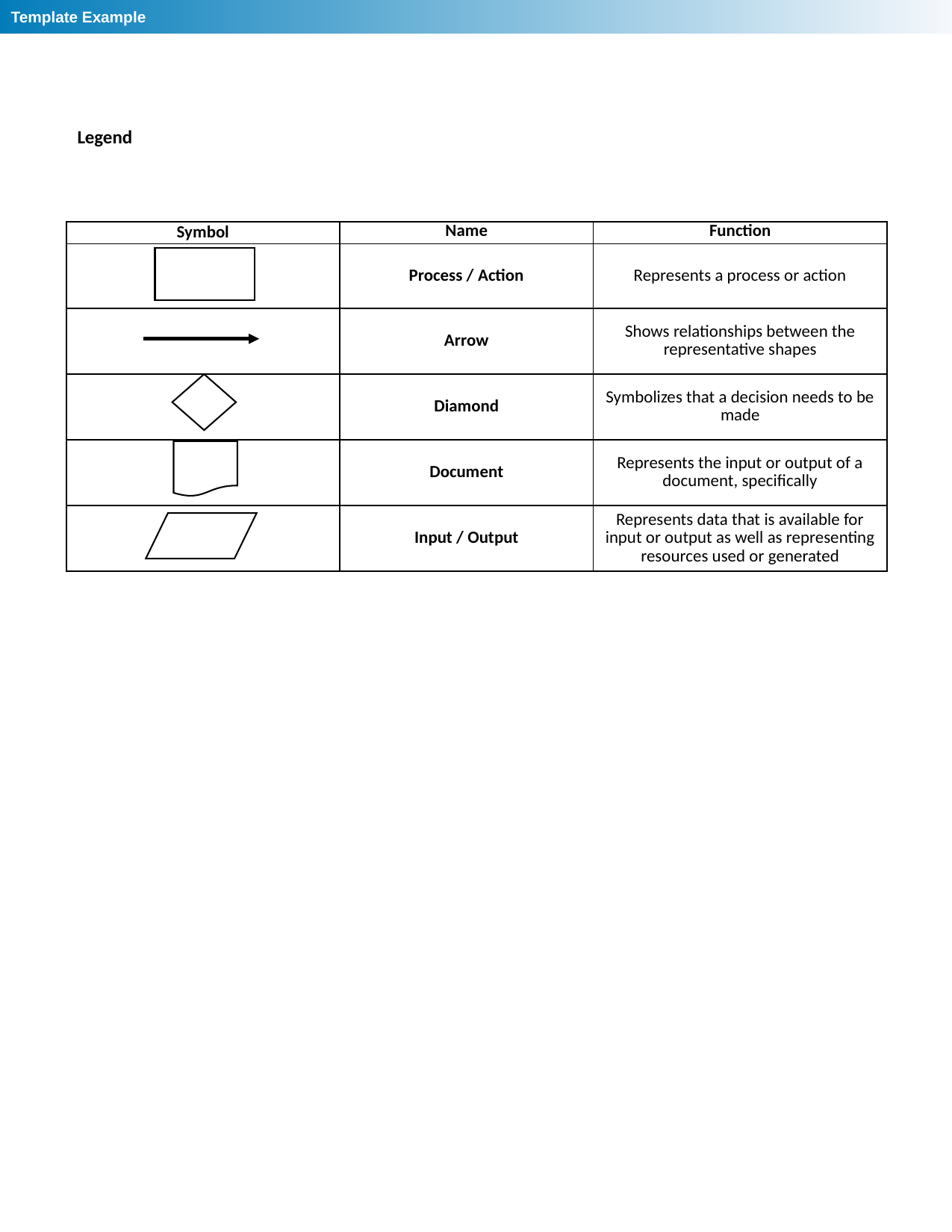

Template Example
Legend
| Symbol | Name | Function |
| --- | --- | --- |
| | Process / Action | Represents a process or action |
| | Arrow | Shows relationships between the representative shapes |
| | Diamond | Symbolizes that a decision needs to be made |
| | Document | Represents the input or output of a document, specifically |
| | Input / Output | Represents data that is available for input or output as well as representing resources used or generated |
