# Supplementary material for: Laboratory quality management system fundamentals
Source: Front Bioeng Biotechnol. 2025 May 21;13:1578654. doi: 10.3389/fbioe.2025.1578654 (PMC12133829; doi:10.3389/fbioe.2025.1578654)
Supplement: Supplementary file 1 [file DataSheet1.zip › Supplementary Materials/TEMPLATE_Performance Indicator Development Form.docx]

**Laboratory Key Performance Indicator (KPI) Development Form**

| KPI Characteristic | Details |
| --- | --- |
| KPI ID# | [Enter ID#] |
| Process Name | [Enter name of the process that the KPI pertains to] |
| Strategic Goal or Objective(s) | [Enter the strategic goal(s) or objective(s) that the laboratory’s overarching organization has established that the KPI is related to] |
| Performance Question | [Enter the performance question that the KPI data to be collected will answer in order to assess achievement of goals/objectives; e.g., “How well is the laboratory meeting testing deadlines?”; “How many batches are tested per month?”] |
| KPI | [Enter a brief description of the KPI; following the example above, “Sample testing completion timeliness.”] |
| Operational Definition | [Enter the specific metric data that will be collected at intervals, to reflect how it will be calculated; following the same example above, “Percent of sample tests completed within the agreed-upon timeframe.”] |
| Formula | [Enter the calculation that the appropriate personnel will use to gather the data at intervals to meet the operational definition; following the same example above, “ % = ( # tests completed by the due date) ÷ (# test orders received/scheduled).”] |
| Target Value | [Enter target value for the data to be gathered; e.g., “95%”] |
| Data Storage Location | [Enter the location that the KPI data will be stored in; e.g., laboratory information management system (LIMS) link; SharePoint library; or network drive link] |
| Reporting Format | [Enter brief description of how the collected data will be presented to stakeholders; e.g., quarterly report as a PowerPoint file delivered via email or in an established meeting] |
| Reporting Period | [Enter the timeframe that the KPI reporting covers; e.g., quarterly, one fiscal year, one calendar year, etc.] |
| Review Frequency | [Enter how often this KPI item will be reviewed for effectiveness and relevance; e.g., annual, triennial, etc.] |

**Revision History**

| Version | Effective Date | Summary of Change(s) Made to Previous Version |
| --- | --- | --- |
| [x.x] | [MM/DD/YYYY] | 1. [Enter change (e.g., “New document”)] |

**Approval**

**Approved by: [Signature]**

____________________________________________

[Title]; [Name of Division/Department/Office, etc.]
