# Supplementary material for: Laboratory quality management system fundamentals
Source: Front Bioeng Biotechnol. 2025 May 21;13:1578654. doi: 10.3389/fbioe.2025.1578654 (PMC12133829; doi:10.3389/fbioe.2025.1578654)
Supplement: Supplementary file 1 [file DataSheet1.zip › Supplementary Materials/TEMPLATE_SOP.docx]

1. Purpose
2. [Provide a brief statement describing the intent of this SOP.]
3. [Provide a brief statement describing the intent of this SOP (continued).]
4. Scope
5. [Describe who and what this SOP will affect.]
6. [Describe who and what this SOP will affect (continued).]
7. background
8. [Optional section. If using, list pertinent context and information that helps new or occasional users understand why the SOP exists. If not using, delete section C. Background]
9. [Pertinent context and information (continued).]
10. responsibilities

## [INSERT ROLE OF RESPONSIBLE PERSON INVOLVED IN THE PROCEDURE (E.G., Laboratory technician, laboratory manager)]

- 1. [List general responsibilities for this role as they relate to this SOP.]
     1. [Insert additional text as needed.]
     2. [Insert additional text as needed.]
  2. [List general responsibilities for this role as they relate to this SOP.]
     1. [Insert additional text as needed.]
     2. [Insert additional text as needed.]

## [INSERT ROLE OF RESPONSIBLE PERSON INVOLVED IN THE PROCEDURE (E.G., Laboratory technician, laboratory manager)]

- 1. [List general responsibilities for this role as they relate to this SOP.]
     1. [Insert additional text as needed.]
     2. [Insert additional text as needed.]
  2. [List general responsibilities for this role as they relate to this SOP.]
     1. [Insert additional text as needed.]
     2. [Insert additional text as needed.]

**Note: As an alternative to completing this section’s steps, author(s) may attach a completed Responsibility Assignment Matrix to the appendix of this SOP (see Appendix A for matrix template), delete placeholder steps above, and use the below text in this section). Delete this text once section completed.*

*See Appendix [x] for responsibility matrix.*

1. DEFINITIONS

| **Term** | **Definition** |
| --- | --- |
| [In case jargon or unusual terms are used, insert here to be defined.] | [Insert the meaning here. Add rows as needed.] |
|  |  |
|  |  |

1. Procedure
2. [INSERT MAIN ACTIVITY HEADING TEXT]
   1. [Provide detailed steps that describe the actions users need to execute.]
      1. [Insert additional sub-step text as needed.]
      2. [Insert additional sub-step text as needed.]
   2. [Provide detailed steps that describe the actions users need to execute.]]
      1. [Insert additional sub-step text as needed.]
      2. [Insert additional sub-step text as needed.]

## [Insert MAIN ACTIVITY HEADING TEXT]

- 1. [Provide detailed steps that describe the actions users need to execute.]]
     1. [Insert additional sub-step text as needed.]
     2. [Insert additional sub-step text as needed.]
  2. [Provide detailed steps that describe the actions users need to execute.]
     1. [Insert additional sub-step text as needed.]
     2. [Insert additional sub-step text as needed.]

1. acronyms & abbreviations

| **Acronym/Abbreviation** | **Definition** |
| --- | --- |
| [Insert acronym/abbreviation to be defined.] | [Insert acronym/abbreviation’s full term here. Add rows as needed.] |
|  |  |
|  |  |

1. references
2. [Enter any applicable reference titles here (and attach or link reference documents to appendix as needed]
3. Original sop Document management

| Original SOP Development Role | Organization & Role, Group, or Program |
| --- | --- |
| Lead Author | [Insert Organization; Role] |
| Owner | [Insert Organization; Group, Program or Role] |
| SMEs/Contributors | [Insert Organization; Group, Program or Role] |
| SOP Users | [Insert Organization; Group(s), Program(s), or Role(s); (e.g., All Laboratory Staff, QC Analysts] |
| SOP Reviewers | [Insert Organization; Group, Program or Role] |
| Approvers | [Insert Role(s) of SOP approvers] |

1. REVISION HISTORY

| Version | Effective Date | Summary of Change(s) Made to Previous Version |
| --- | --- | --- |
| [x.x] | [MM/DD/YYYY] | 1. [Describe the change made to the previous version of the SOP (e.g., N/A - New controlled document; Section D: links updated)] 2. [Describe additional change, as needed.] |

1. approval

| Organizational Role | Name | Reviewer/Approver | Signature and Date |
| --- | --- | --- | --- |
| [Title], [Name of Division/Branch/Office, etc.] | [First and Last Name (add rows, as needed)] | [Insert “Reviewer” or “Approver”] |  |
|  | [First and Last Name] | [Insert “Reviewer” or “Approver”] |  |
|  | [First and Last Name] | [Insert “Reviewer” or “Approver”] |  |
| [Title], [Name of Division/Branch/Office, etc.] | [First and Last Name] | [Insert “Reviewer” or “Approver”] |  |

# Appendix A: Responsibility – Accountability – Support – Consulted – Informed (RASCI) Chart (*Optional template – delete if not using*)

| ***RASCI Chart for [Insert SOP Name]** | | | | | | | | |
| --- | --- | --- | --- | --- | --- | --- | --- | --- |
| **ROLES** | | [Staff Role Involved] | [Staff Role Involved] | [Staff Role Involved] | [Staff Role Involved] | Organization/Role Involved | Organization/Role Involved |  |
| **Item #** | **Major Process Activity/Task** | **FTEs** | | **Part-Time Employees or Contractors** | | **External Parties** | |  |
|  |  |  |  |  |  |  |  |  |
|  |  |  |  |  |  |  |  |  |
|  |  |  |  |  |  |  |  |  |
|  |  |  |  |  |  |  |  |  |
|  |  |  |  |  |  |  |  |  |
|  |  |  |  |  |  |  |  |  |
|  |  |  |  |  |  |  |  |  |
|  |  |  |  |  |  |  |  |  |
|  |  |  |  |  |  |  |  |  |
|  |  |  |  |  |  |  |  |  |
|  |  |  |  |  |  |  |  |  |
| *"Responsibility – Accountability – Support – Consulted – Informed" Chart Key | | | | | | | | |
| **R** | Responsible | Assigned to complete the task | | | | | |  |
| **A** | Accountable | Has final decision-making authority and accountability for completion of task | | | | | |  |
| **S** | Support | Provides task support | | | | | |  |
| **C** | Consulted | An advisor/stakeholder/subject matter expert who is consulted for task decisions | | | | | |  |
| **I** | Informed | Must be informed after a task-related decision | | | | | |  |

# Appendix B: [Name of Appendix or Attachment (as needed)]
